# Central hyperthyroidism due to an ectopic TSH-secreting pituitary tumor: a case report and literature review

**DOI:** 10.3389/fendo.2024.1301260

**Published:** 2024-03-07

**Authors:** Chonggui Zhu, Tong Liu, Haonan Yu, Lina Chang, Xiaona Zhang, Jia Yao, Geng Zhang, Qiusong Chen, Qing He, Ming Liu

**Affiliations:** ^1^ Department of Endocrinology and Metabolism, Tianjin Medical University General Hospital, Tianjin, China; ^2^ Department of Positron Emission Tomography/Computed Tomography (PET/CT) Examination Room, Tianjin Medical University General Hospital, Tianjin, China; ^3^ Department of Radiology, Tianjin Medical University General Hospital, Tianjin, China; ^4^ Department of Otorhinolaryngology, Tianjin Medical University General Hospital, Tianjin, China

**Keywords:** TSH-secreting tumor, ectopic pituitary tumor, diagnosis, octreotide suppression test, 68 Ga-DOTATATE PET/CT

## Abstract

Ectopic thyroid-stimulating hormone (TSH)-secreting tumors are extremely rare, with only 15 reported cases in the literature. Herein, we described a 60-year-old female patient with thyrotoxicosis and elevated or unsuppressed levels of TSH. Family history and laboratory and genetic tests did not support a diagnosis of resistance to thyroid hormone (RTH). Given the unsuppressed TSH, TSH-secreting tumor was suspected, and magnetic resonance imaging (MRI) of the pituitary gland was performed. Surprisingly, the MRI scans revealed a nodule in the nasopharynx rather than a pituitary tumor in the sella region. Further evaluation using Gallium-68 DOTATATE positron emission tomography/computed tomography (^68^Ga-DOTATATE PET/CT) demonstrated increased DOTATATE uptake in the nasopharyngeal nodule. Additionally, an octreotide suppression test (OST) revealed an obvious reduction in TSH levels, further supporting the suspicion of the nasopharyngeal mass as the cause of inappropriate TSH secretion. To prepare for surgery, the patient received preoperative administration of octreotide, resulting in the normalization of TSH and thyroid hormone levels. The patient subsequently underwent successful surgical removal of the nasopharyngeal mass. Following the procedure, the patient experienced complete resolution of hyperthyroidism symptoms, with TSH declined and thyroid hormone levels returned to normal. Histochemistry analysis of the tumor revealed positive staining for TSH, growth hormone (GH), prolactin (PRL), luteinizing hormone (LH), and somatostatin receptor 2 (SSTR2). We discussed differential diagnosis of hyperthyroidism due to inappropriate TSH secretion, with a particular emphasis on the importance of ^68^Ga-DOTATATE PET/CT in combination with OST for identifying ectopic pituitary tumors.

## Introduction

Thyroid-stimulating hormone (TSH)-secreting adenoma is a rare type of pituitary tumor, accounting for <3% of all pituitary tumors (1, 2). Therefore, ectopic TSH-secreting pituitary adenomas causing hyperthyroidism are even rare. Since the first reported case in 1996, only 15 cases have been documented in the literatures (3–17). In some occasions, the differential diagnosis between TSH-secreting tumor and pituitary resistance to thyroid hormone action (PRTH) can be difficult, especially when the absence of a TSH-secreting tumor in the pituitary gland is recognized. Therefore, the establishment of the ectopic TSH-secreting tumor is often challenging. In this report, we described a case of ectopic TSH-secreting adenoma at nasopharynx. Next, we reviewed previous cases of ectopic TSH-secreting adenomas reported in the literature and summarized the diagnostic process and management of this rare disease.

## Case presentation

A 60-year-old woman with a complaint of weight loss, palpitation, tremor, and heat intolerance was admitted to Endocrinology and Metabolism Department of the Tianjin Medical University General Hospital due to abnormal thyroid function tests. The results exhibited normal levels of TSH but elevated free thyroxine (FT4) and free triiodothyronine (FT3). The diagnosis of hyperthyroidism and thyroid nodules were established 2 years ago based on her abnormal thyroid function tests results and thyroid ultrasound examination, while no antithyroid drugs were prescribed. Recently, the patient’s symptoms of hyperthyroidism worsened, and subsequent thyroid function tests continued to indicate hyperthyroidism. During hospitalization, she also presented tachycardia, with a heart rate of 110 beats per minute, but showed no signs of exophthalmos or pretibial myxedema. The palpable thyroid gland was enlarged, and bilateral hand resting tremors were notable upon physical examination. There was no family history of thyroid diseases.

Thyroid function tests were repeated, revealing elevated levels of TSH (5.099 μIU/ml; reference, 0.350–4.940), FT4 (29.61 pmol/L; reference, 9.01–19.05), and FT3 (17.15 pmol/L; reference, 2.63–5.70). Thyroid peroxidase antibody, thyroglobulin antibody, and TSH receptor antibody were all within the normal range. Thyroid nodules were classified as TIRADS 2 or 3, and a slightly enlarged thyroid gland was observed on thyroid ultrasonography. An increased tracer uptake in the right thyroid gland was showed in thyroid emission computed tomography (ECT) scan. Pituitary hormones, including adrenocorticotropic hormone (ACTH), GH, PRL, cortisol, and insulin-like growth factor 1 (IGF-1), were within the normal range. However, both luteinizing hormone (LH) and follicle-stimulating hormone (FSH) levels were elevated, along with a low estradiol level. The level of sex hormone-binding globulin (SHBG) was 205 nmol/L (19.0–117.0), and the bone metabolic markers, including C-terminal cross-linked telopeptide (CTX, 1.65 ng/mL; reference, 0.55–1.01), osteocalcin (51.48 ng/mL; reference, 11.00–48.00), and N-terminal propeptide of type 1 procollagen (P1NP, 144.9 ng/mL; reference, 19.00–84.00), were elevated. Her son’s thyroid function tests were within the normal range, and a genetic test for TR-β mutation was negative.

**Figure 1:**
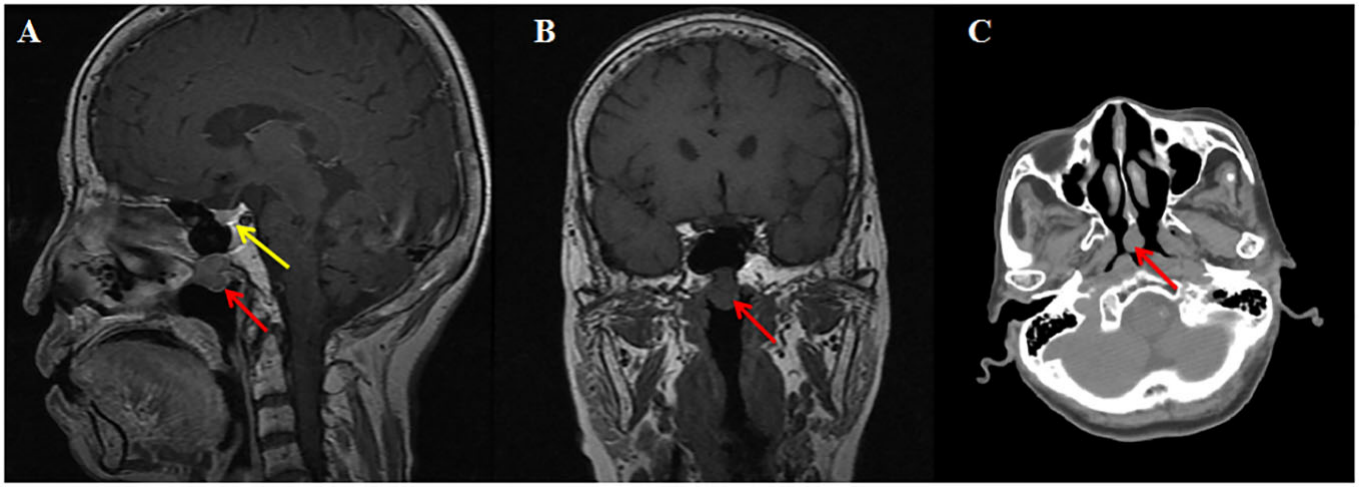
Imaging of MRI and CT. T1-weighted sagittal **(A)** and coronal **(B)** magnetic resonance imaging reveal a mildly enhancing nasopharyngeal nodule (red arrow) and normal pituitary (yellow arrow). Computed tomography **(C)** shows a nasopharyngeal soft tissue nodule.

**Figure 2:**
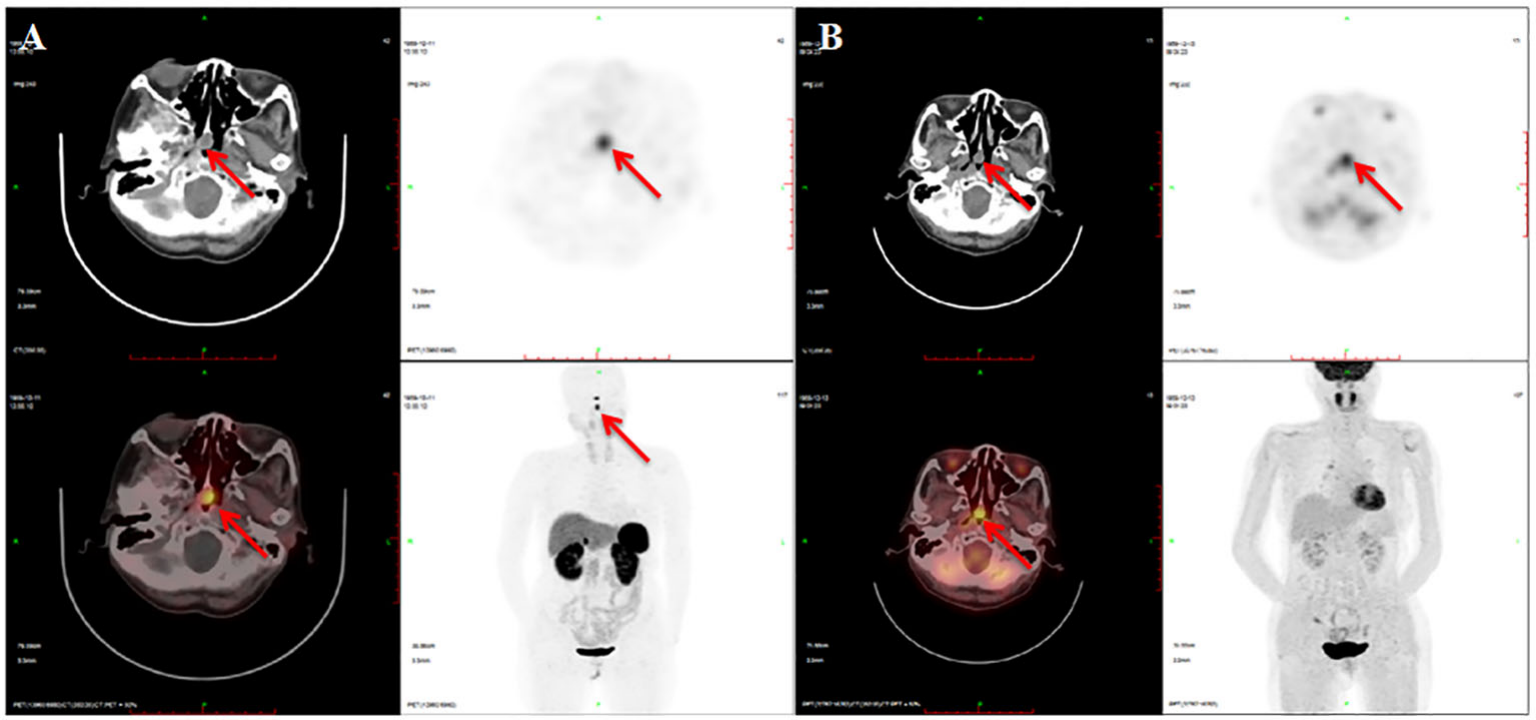
Imaging of ^68^Ga-DOTATATE PET/CT and ^18^F-FDG PET/CT. There is a soft tissue nodule at the apex of the nasopharynx with ^68^Ga-DOTATATE **(A)** and ^18^F-FDG **(B)** intense uptake.

Next, a suppression test was performed by administering 0.1 mg somatostatin analog octreotide at 8:00, and a calculated decline of 60.5% in TSH level was observed on the 16th hour, indicating the possibility of a pituitary TSH-secreting tumor. However, a negative pituitary MRI ruled out the presence of pituitary TSH-oma as the cause of central hyperthyroidism in this patient. Surprisingly, a mass measuring approximately 13 mm × 11 mm was discovered in the nasopharynx on the pituitary MRI, which is consistent with the nasopharyngeal soft tissue nodule presented on paranasal sinus computed tomography (CT) (**Figure 1**). Therefore, a probable ectopic TSH-secreting tumor of the nasopharynx was suspected. To further investigate, an ^18^F-fluorodeoxyglucose positron emission tomography/computed tomography (^18^F-FDG PET/CT) was performed, revealing increased glucose uptake in the nasopharyngeal mass (**Figure 2**). To confirm a neuroendocrine tumor, an additional ^68^Ga-DOTATATE PET/CT was performed, which showed a significance tracer uptake in the same lesion (**Figure 2**), corresponding to the result of the OST. Based on these findings, an ectopic TSH-secreting tumor of the nasopharynx was considered. Consequently, surgical removal of the mass was planned and prepared by administering 0.1 mg of octreotide injection three times per day for 7 days, resulting in a decline in TSH level from 3.494 to 0.049 μIU/mL and normalization of FT3 and FT4 levels.

**Figure 3:**
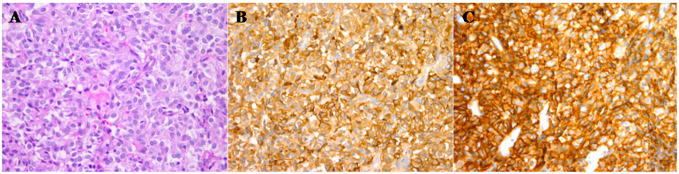
Pathological and immunohistochemical staining results of tumors. Under the microscope, the tumor is composed of sheets of uniform small cells with slightly eosinophilic cytoplasm and round to oval nuclei (hematoxylin and eosin, ×400). Some tumor cells arrange in a pseudorosette pattern **(A)**. Immunohistochemistry stain (×400) demonstrates diffuse and strong reactivities against both TSH **(B)** and SSTR2 **(C)**.

The otolaryngologist successfully performed endoscopic resection of the mass. Pathological and immunohistochemical staining examinations confirmed the presence of an ectopic pituitary neoplasm. The tumor cells were positive for TSH, GH, PRL, LH, SSTR2, pituitary-specific POU-class homeodomain transcription factor (PIT-1), and estrogen receptor alpha (ERα), and negative for FSH, ACTH, transcription factor19 (T-PIT), and steroidogenic factor-1 (SF-1) (**Figure 3**). The Ki-67 index was 0.5%.

On the seventh day after surgery, the patient had a high FT3 value of 5.75 pmol/L (2.63–5.70), while FT4 and TSH values were within normal range. Her initial symptoms were partially recovered at 6 months postoperatively, but her hyperthyroidism recurred and TSH level increased to 4.69 μIU/ml (0.35–5.1). Hence, she was readmitted for a second assessment of the tumor. Thyroid function tests showed that TSH was 3.681 μIU/mL (0.350–4.940), FT4 was 31.91 pmol/L (9.01–19.05), and FT3 was 14.69 pmol/L (2.63–5.70). The OST showed a 79.5% decline in TSH levels at the 16th hour after the drug injection. The nasopharynx MRI and ^68^Ga-DOTATATE PET/CT showed similar results as the first admission. After multidisciplinary discussion, the remnant mass was removed, resulting in a decline in TSH level to <0.004 μIU/mL, with FT3 and FT4 values within normal range. A thyroid function test conducted in May 2021, 5 months later, showed TSH value of 0.022 (0.51–4.78), with FT3 and FT4 remaining normal.

## Discussion

Ectopic TSH-secreting pituitary adenoma is an extremely rare disease characterized by a pituitary adenoma located outside the sella turcica and normal presentation of intrasellar pituitary gland (2). Diagnostic and therapeutic processes can be challenging due to the lack of guidelines and recommendations. However, there have been an increasing number of reported cases of TSH-secreting tumor in recent years. Our case is the 16th reported case of this rare disease, and previous 15 cases have been summarized in **Supplementary Table S1**. Although the exact mechanisms underlying ectopic TSH-secreting tumor have not been fully understood, the theory of the embryogenesis has been proposed to elucidate this rare disease (14). The adenoma with ectopic hormonal production derived from the remnants of embryonic cells migrates through the craniopharyngeal canal to the sphenoid sinus or nasopharynx (14).

Most previous cases were misdiagnosed as Graves’ disease, treated with antithyroid drugs or iodine-131. Even levothyroxine was given to patients with the misdiagnosis of primary hypothyroidism based on the elevation of TSH (14). The unusual phenomenon of inappropriate TSH secretion often suggests rarely clinical disorders rather than Graves’ disease. It is important to consider potential interferences in TSH assays whenever there are unexplained thyroid function test results. Various substances, such as macro-TSH, biotin, antistreptavidin antibodies, anti-ruthenium antibodies, and heterophilic antibodies, can affect TSH measurement. These interferences can be identified by a series of laboratory tests, such as assay method comparison, dilution procedures, blocking reagents studies, and polyethylene glycol precipitation (18). Unfortunately, these laboratory tests were not performed in our case due to technical limitations. However, the possibility of interferences in TSH measurements was excluded based on the patient’s clinical manifestations and repeated thyroid function tests conducted in different laboratories.

The primary differential diagnosis in this case is resistance to thyroid hormone (RTH), specifically peripheral resistance to thyroid hormone (PRTH), which has been previously described in case reports (7, 13, 14). RTH is characterized by reduced sensitivity to thyroid hormone and is caused by mutations in the thyroid hormone receptor β gene, resulting in intranuclear T3 receptor dysfunction. The hallmark symptoms of RTH include goiter, sinus tachycardia, attention-deficit hyperactivity disorder, elevated levels of FT3 and FT4, and inappropriate TSH secretion (19). Differential diagnosis is crucial in cases of hyperthyroidism with elevated TSH. Sometimes the differentiation is a challenge especially when ectopic TSH-secreting tumor, RTH with non-functioning pituitary adenoma, TSH-secreting pituitary adenoma, or RTH co-exist (20, 21). In brief, RTH is a rare autosomal dominant disease that can be diagnosed by positive mutation in TRβ, positive family history, age at onset, goiter, and other clinical manifestations (22). Negative family history and genetic testing for TRβ mutation in our case enabled us to distinguish between two conditions. Additionally, in hyperthyroidism caused by TSH-secreting tumors, peripheral tissues exhibit sensitivity to thyroid hormone, and certain serum markers can be helpful in diagnosis. Increased serum concentrations of sex hormone-binding globulin (SHBG) and bone turnover markers, and elevated α-GSU/TSH molar ratio, could indicate the presence of TSH-secreting tumors (13, 23). In addition, some tumors are plurihormonal, expressing GH and/or PRL along with TSH; hence, the measurement of GH and PRL can be helpful in diagnosing TSH-secreting tumor. Interestingly, in our case, serum GH and PRL levels were within the normal range, but immunohistochemical staining examinations revealed positive TSH, GH, and PRL expression in tumor cells. Furthermore, dynamic function tests including TRH stimulation test, T3 suppression test, and OST can be used to assist in confirming the diagnosis (23, 24). Literature reports indicate that a diminished TSH response to TRH stimulation is observed in 96% of TSH-secreting tumors, while TRH test stimulates TSH secretion in 97% of RTH cases (24). In patients with TSH-secreting tumors, incomplete suppression can be observed during the T3 suppression test (25). Unfortunately, due to the lack of TRH and T3, these two significant tests were not conducted. Although OST is not recommended in current guidelines, it has been used as an effective tool to differentiate between TSH-secreting tumors and RTH. Liu et al. reported a maximal decrease in TSH levels of 89.07% (73.85%, 96.77%) in TSH-secreting pituitary adenomas during the entire OST (23, 26, 27). In our case, the TSH suppression rates during two OSTs were 60.5% and 79.5%, respectively. Considering the other examination results, we highly suspect the presence of an ectopic TSH-secreting tumor.

It is unable to distinguish ectopic TSH-secreting tumors from intrasellar tumors by clinical and biochemical methods, with the exception of pituitary gland MRI. Ectopic TSH-secreting tumors and TSH-secreting pituitary adenoma exhibit similar clinical manifestations of central hyperthyroidism, such as palpitation, weight loss, heat in tolerance, and thyroid goiter. In some cases, pituitary adenomas can cause hypopituitarism and associated symptoms, while ectopic tumors can present with ear, nose, and visual symptoms, among others (8, 17). Among the previous reported cases of ectopic TSH-secreting tumors, 10 cases were found to be located in the nasopharynx (**Supplementary Table S1**). Clinically, symptoms of nasal obstruction may provide value clues in suggesting the presence of a nasopharyngeal mass during the diagnostic process of ectopic TSH-secreting tumors. Nine of 15 cases (60%) presented symptoms of nasopharyngeal tissue compression. However, the typical symptoms of nasal obstruction are often unnoticed until the occurrence of a nasopharyngeal mass.

Determining the neuroendocrine origin of nasopharyngeal mass is crucial for diagnostic and treatment strategies. Tumor biopsy, although a choice, was not performed due to its invasiveness. Recently, functional imaging with PET tracers has emerged as a valuable tool for detecting and diagnosing neuroendocrine tumors (28, 29). In the case of TSH-secreting tumors, tumor cells typically express abundant somatostatin receptor subtype (SSTR) 2 and 5 on their surface. Therefore, ^68^Ga-DOTATATE or ^68^Ga-DOTANOC PET/CT has been demonstrated to be effective in the diagnosing ectopic TSH-secreting tumor, especially when distinguishing them from intrasellar adenoma (12, 13). Case 10 was the first to report the use of ^68^Ga-DOTATATE PET/CT for localizing the ectopic TSH-secreting tumor (12). Fortunately, ^68^Ga-DOTATATE PET/CT confirmed that the nasopharyngeal mass in this case was a neuroendocrine tumor, possibly a TSH-secreting tumor with somatostatin receptor expression, which was hypermetabolically shown in ^18^F-FDG PET/CT. Although conventional methods such as ^18^F-FDG PET/CT or 99-mTc octreotide scan played an important role in the diagnostic process of previous case reports, somatostatin receptor imaging has proven to be a superior method for detecting and diagnosing ectopic TSH-secreting tumors (12, 13). In case 14, positron emission tomography/magnetic resonance imaging (PET/MRI) using ^65^Ga-labeled octreotide and ^18^F-labeled FDG as markers successfully contributed to the diagnosis (16). However, ^68^Ga-DOTATATE PET/CT may play a more important role in lesion detection of neuroendocrine tumors compared to Octreoscan, ^18^F-FDG PET/CT, CT, and MRI (30). Furthermore, the use of ^68^Ga-DOTATATE or ^68^Ga-DOTANOC PET/CT is expensive and should be reserved for cases with a high suspicion of ectopic neuroendocrine tumors. Therefore, positive OST results not only support the diagnosis of a TSH-secreting tumor but also determine the need for ^68^Ga-DOTATATE PET/CT imaging.

All the patients with this rare disease underwent surgery because transsphenoidal or subfrontal adenomectomy is the first-line therapy for TSH-secreting tumors (23). Prior to surgery, our patient received a dosage of 0.1 mg octreotide injections every 8 h for 1 day, which effectively normalized their FT3 and FT4 levels. This preoperative preparation method is also recommended by guidelines (13, 23). The administration of octreotide for a duration of 5–7 days can adequately prepare patients for surgery without complications of hyperthyroidism, and it is also recommended that a dose of sustained-release octreotide can be used as preoperative treatment for patients with severe complications such as atrial fibrillation. Anti-thyroid drugs are generally considered the second-line treatment option and are often used in patients who do not respond well to somatostatin (16). Methimazole can be prescribed for patients with a coexistence of TSH-secreting tumor and Graves’ disease (31).

In our case, the patient underwent two consecutive surgeries, and thyroid function results are currently within the normal range. The criteria for determining the cure of TSH-secreting tumors have not been clearly established (23). However, the criteria for cure should include clinical remission of hyperthyroidism, normalization of thyroid hormones or biochemical markers, and resolution of neuroradiological abnormalities. In fact, complete suppression of TSH secretion in the T3 suppression test may be the best indicator for evaluating the success of lesion removal (32). Since there are no available criteria for determining a cure in ectopic TSH-secreting tumors, long-term follow-up and regular biochemical and radiological examinations are crucial for managing this rare disease, especially in the case of our patient.

## Conclusion

A female patient presenting with central hyperthyroidism was diagnosed with a rare ectopic TSH-secreting tumor located in the nasopharynx, which is a frequently observed site for such lesions. The combination of an octreotide suppression test and ^68^Ga-DOTATATE PET/CT can be a valuable tool for the identification of ectopic pituitary tumors. Finally, this rare disease should be taken into consideration by endocrinologists when encountering cases of hyperthyroidism with normal or elevated TSH.

## Data availability statement

The raw data supporting the conclusions of this article will be made available by the authors, without undue reservation.

## Ethics statement

Written informed consent was obtained from the individual(s) for the publication of any potentially identifiable images or data included in this article.

## Author contributions

CZ: Data curation, Writing – original draft, Writing – review & editing. TL: Writing – original draft, Writing – review & editing. HY: Visualization, Writing – original draft, Writing – review & editing. LC: Formal analysis, Writing – review & editing. XZ: Data curation, Supervision, Writing – review & editing. JY: Project administration, Visualization, Writing – review & editing. GZ: Project administration, Visualization, Writing – review & editing. QC: Visualization, Writing – review & editing. QH: Investigation, Resources, Supervision, Writing – review & editing. ML: Funding acquisition, Resources, Supervision, Validation, Writing – review & editing.
