# Supplementary material for: Central hyperthyroidism due to an ectopic TSH-secreting pituitary tumor: a case report and literature review
Source: Front Endocrinol (Lausanne). 2024 Mar 7;15:1301260. doi: 10.3389/fendo.2024.1301260 (PMC10955116; doi:10.3389/fendo.2024.1301260)
Supplement: Supplementary file 1 [file Table_1.docx]

**Supplemental TableS1.** Clinical characteristics of published cases of ectopic TSH-secreting tumors and the present case report

| Case/Reference number | Age/Gender | Location | Octreotide suppression test | Imaging | Functional Imaging | Application of somatostatin  analogs for surgery | Immunohistochemistry |
| --- | --- | --- | --- | --- | --- | --- | --- |
| 1/[3] | 66/F | nasopharynx | - | CT,MRI | - | - | TSH(+)，GH(+)，PRL(+)，FSH(+)，ACTH(+)，LH(+) |
| 2/[4] | 52/M | nasopharynx | - | MRI | - | - | TSH(+) |
| 3/[5] | 50/F | nasopharynx | - | CT,MRI | - | - | TSH(+)，GH(+)，FSH(+)  LH(+) |
| 4/[6] | 49/F | nasopharynx | positive | CT,MRI | 99mTc-labeled  octreotide scan(+) | 0.1mg octreotide subcutaneous injection every 8h for 7 days | TSH(+)，GH(+)，PRL(+) |
| 5/[7] | 41/M | nasopharynx | positive | CT,MRI | - | - | TSH(+)，GH(+) |
| 6/[8] | 46/M | nasopharynx | - | CT,MRI | positron emission tomography imaging(+) | - | TSH(+) |
| 7/[9] | 46/M | suprasellar space | - | MRI | - | octreotide for three days | TSH(+) |
| 8/[10] | 37/F | nasopharynx | - | MRI | - | - | TSH(+) |
| 9/[11] | 41/M | suprasellar | - | CT,MRI | ^18^F-FDG PET(-) | - | TSH(+) |
| 10/[12] | 48/F | nasopharynx | - | MRI | ^68^Ga-DOTATATE PET/CT(+) | - | TSH(+)，PRL(+) |
| 11/[13] | 48/F | nasopharynx | - | MRI | ^18^F-F-DOPA PET-CT(-)  ^68^Ga-DOTANOC PET/CT(+) | 0.1mg of octreotide three  times daily over 5 days | TSH(+)  LH(+) |
| 12/[14] | 52/F | sphenoid  sinus | positive | CT,MRI | - | a single intramuscular dose of 20mg-sustained-release  somatostatin | TSH(+)，GH(+) |
| 13/[15] | 37/M | nasopharynx | - | MRI | - | Octreotide treatment was utilized in pre-operative, but without details in the article | TSH(+)，GH(+)。PRL(+) |
| 14/[16] | 10/F | Suprasellar Region | positive | MRI | PET/MRI with  ^65^Ga-labeled octreotide and ^18^F-labeled FDG as markers | 0.1 mg octreotide  subcutaneous injection for 4 days | TSH(+)，GH(+)，ACTH(+)  LH(+)，FSH(+)，SSTR2(+) |
| 15/[17] | 71/F | sphenoid bone | - | CT,MRI | - | - | TSH(+)，GH( +)，PRL( +) |
| Current report | 60/F | nasopharynx | positive | CT,MRI | ^18^F-FDG PET/CT(+)  ^68^Ga-DOTATATE PET/CT(+) | 0.1mg of octreotide injection three times one day for 7days | TSH(+)，GH(+)，PRL(+)，  LH(+) ，SSTR2(+) |

F: female；M: male
